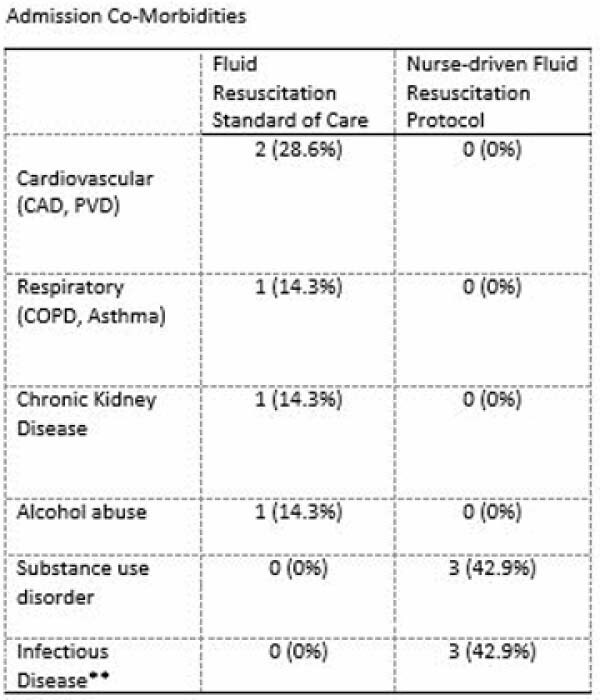# 526 Results of Implementation of a Nurse Driven Fluid Resuscitation Protocol in a Regional Burn Center

**DOI:** 10.1093/jbcr/irad045.123

**Published:** 2023-05-15

**Authors:** Emily Werthman, Julie Caffrey, Carrie Cox

**Affiliations:** Johns Hopkins Bayview Medical Center, Lutherville, Maryland; Johns Hopkins University School of Medicine, Baltimore, Maryland; Johns Hopkins Adult Burn Center, Baltimore, Maryland; Johns Hopkins Bayview Medical Center, Lutherville, Maryland; Johns Hopkins University School of Medicine, Baltimore, Maryland; Johns Hopkins Adult Burn Center, Baltimore, Maryland; Johns Hopkins Bayview Medical Center, Lutherville, Maryland; Johns Hopkins University School of Medicine, Baltimore, Maryland; Johns Hopkins Adult Burn Center, Baltimore, Maryland

## Abstract

**Introduction:**

Our prior research has demonstrated the effect of nurse driven fluid resuscitation on provider and nurse team dynamics. Our primary purpose with this new research on our nurse driven fluid resuscitation protocol is to determine the effect of the nurse-driven fluid resuscitation protocol on resuscitation associated complications (i.e., acute respiratory distress syndrome (ARDS), acute kidney injury (AKI), and abdominal compartment syndrome). The study hypothesis is that the rate of resuscitation associated complications will remain stable or not increase post-protocol implementation.

**Methods:**

Retrospective review for all patients within a one-year period pre-protocol implementation and post-protocol implementation was completed. 44 patients were included in the total sample, 22 in the pre-implementation group and 22 in post-implementation group. Exclusion criteria for patients included in nurse driven fluid resuscitation included delayed presentation, morbid obesity, electrical injury, cardiac history, DKA, polytrauma, and renal failure.

**Results:**

Pre and post nurse driven fluid resuscitation implementation samples of 22 patients with total body surface area (TBSA) greater than 20% were analyzed. The incidence of serious complications including ARDS, compartment syndrome, and wound conversion was not statistically significantly different between samples. Rates of acute kidney injuries were similar across groups. In addition, the mean and median TBSA were not statistically significantly different pre and post implementation. The length of stay for the pre-implementation sample was on average longer than for the post-implementation sample. Our results are limited by the significant comorbidities of the pre-implementation group.

Please note tables and graphs were not yet available by time of submission. Attached tables are from prior analysis at approximate half-way of study. Updated graphs and tables will be complete by conference time.

**Conclusions:**

As hypothesized, there were no differences in outcomes between patients receiving nurse driven fluid resuscitation. Our results indicate that nurse driven fluid resuscitation is a safe and clinically effective practice in the burn center. Coupled with our previous results that indicate a significant increase in nurse satisfaction with interdisciplinary communication associated with nurse driven, these results signal the importance of implementing nurse led resuscitations.

**Applicability of Research to Practice:**

Nurse driven fluid resuscitation is a safe and effective. Further research into barriers to implementation is warranted.